# Low-level light therapy using a helmet-type device for the treatment of androgenetic alopecia: A 16-week, multicenter, randomized, double-blind, sham device-controlled trial

**DOI:** 10.1097/MD.0000000000021181

**Published:** 2020-07-17

**Authors:** Jung Soo Yoon, Won Young Ku, Jang Hyun Lee, Hee Chang Ahn

**Affiliations:** a Department of Plastic and Reconstructive Surgery, DongGuk University Ilsan Medical Center, Goyang, South Korea; b Department of Plastic and Reconstructive Surgery, Hanyang University Medical Center, Hanyang University College of Medicine, Seoul; c Department of Plastic and Reconstructive Surgery, Hanyang University Guri Hospital, Hanyang University College of Medicine, Guri, Korea.

**Keywords:** androgenetic alopecia, efficacy, low-level light therapy, randomized double-blind clinical trial, safety

## Abstract

**Introduction:**

Androgenetic alopecia is the most common form of hair loss in both sexes. In recent studies, low-level light therapy (LLLT) has been established as an effective treatment for alopecia. The purpose of this study was to evaluate the safety and efficacy of LLLT using a new helmet-type device for the treatment of androgenetic alopecia.

**Method:**

A randomized, sham device-controlled, double-blind clinical trial was conducted at 2 institutions. Sixty participants diagnosed with androgenetic alopecia aged from 19 to 65 years were recruited. LLLT was performed through a helmet-type device that emitted light with a mean output power of 2.36 mW/cm^2^ at a wavelength of 655 nm. Participants were divided into 2 groups, which respectively used the experimental device and a sham device. After tattooing at the central point of the vertex, phototrichograms at that point were obtained at 0, 8, and 16 weeks. The primary endpoint of the study was the difference in the rate of change of hair density between the test group and the control group.

**Results:**

Comparing the results at baseline and week 16, the experimental group showed an increase in hair density of 41.90 hairs/cm^2^ and an increase in hair thickness of 7.50 μm, whereas the control group showed an increase of 0.72 hairs/cm^2^ and a decrease of 15.03 μm, respectively (*P* < .001). No adverse events or side effects occurred.

**Conclusion:**

LLLT showed a significant effect on increasing hair density in patients with androgenetic alopecia. LLLT could be a safe and effective treatment for androgenetic alopecia in both sexes.

## 1 Introduction

One's hair is a very important component of how 1 makes a personal impression on others. Therefore, hair loss not only affects one's aesthetic appearance, but also has a significant psychological impact, often resulting in deteriorated quality of life. Androgenetic alopecia, also known as male pattern hair loss and female pattern hair loss, is the most common form of hair loss in both sexes, and its prevalence increases with age.^[1–3]^ The current treatment for alopecia is limited to 5-a reductase inhibitors or topical minoxidil, and in severe cases hair transplantation is the only curative therapy.^[4,5]^ However, patients who suffer from side effects or are unresponsive to those therapies have restricted options in terms of alternative therapies.^[4–6]^

Recently, low-level light therapy (LLLT) has been proven to be an effective non-thermal treatment for various dermatologic disorders through its photobiomodulatory effect.^[7–9]^ Furthermore, home devices with lasers and light sources for dermatologic pathologies, such as hair loss and aging skin, are becoming more popular due to their low cost and convenience.^[10]^ Andre Mester first found that low energy 694-nm wavelength lasers induced hair regrowth in mice in 1967.^[11]^ Since then, although the mechanism of action has not been clearly identified, several studies have provided strong evidence that LLLT stimulates hair growth.^[12–16]^

However, the effectiveness and safety of helmet-type LLLT home devices for alopecia remain controversial. A new helmet-type LLLT model (HAIRUP; Y & J Bio, Seoul, Korea) that combines laser diodes (LDs) and light-emitting diodes (LEDs) at a wave length of 655 nm has been developed. The proposal of this study was to verify the efficiency and safety of this helmet-type LLLT device for androgenic alopecia in both sexes.

## 2 Methods

### 2.1 Study design

We designed a randomized, sham device-controlled, double-blind clinical trial at 2 institutions. In total, 60 candidates (40 from Hanyang University Medical Center and 20 from Hanyang University Guri Hospital) voluntarily agreed to participate in the clinical trial and provided written informed consent before the trial. The selected cases were randomized into an experimental and control group and each group used the experimental device or a sham device for 16 weeks. This study was conducted in conformity with the World Medical Association Declaration of Helsinki, and the protocol was approved by the Institutional Review Board of Hanyang University Medical Center (2018–04–028).

### 2.2 Patient enrollment

This study included men and women aged 19 to 65 years with active androgenetic alopecia (Norwood-Hamilton classification of II (IIa) to V for men, Ludwig classification of I to II for women).^[3]^ Principal investigators at both institutions enrolled the participants. The patients involved in this study were required to maintain the same hairstyle during the clinical trial period and to avoid using special hair products and engaging in hair management or manipulation during the clinical trial period. We excluded patients who used other topical or systematic medications for alopecia, who underwent surgical interventions such as hair transplantation, and who had any other disorders affecting the outcomes of the trial (Table 1).

**Table 1:**
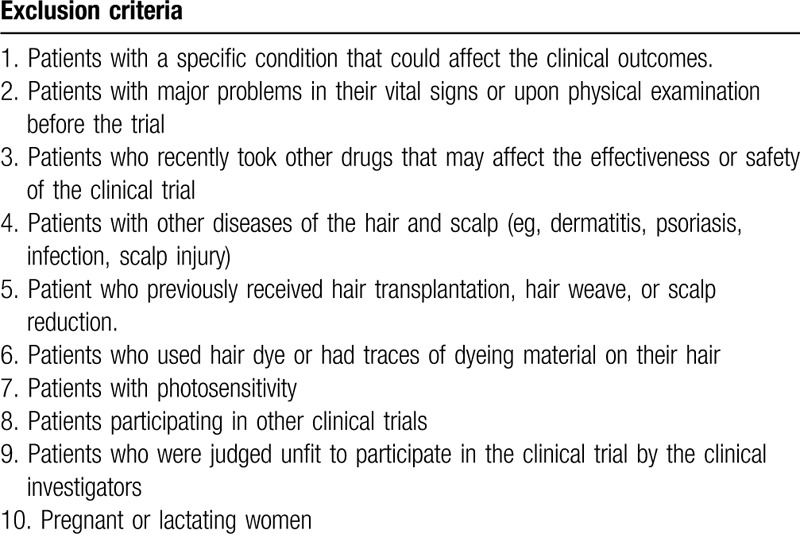
Exclusion criteria.

### 2.3 Randomization and masking

The selected cases were randomized into an experimental and control group by the Medical Statistics Consulting Department, by generating random sequence using the complete randomization method (PASS 14 (Power Analysis and Sample Size Software 2015. NCSS, LLC. Kaysville, Utah). And each group used the experimental devices or a sham devices which were contained in a set of sealed boxes only recorded with a serial number in order of registration. Throughout the study, both the participants and the clinical investigators maintained their double-blind status.

### 2.4 Intervention (experimental and sham devices)

**Figure 1:**
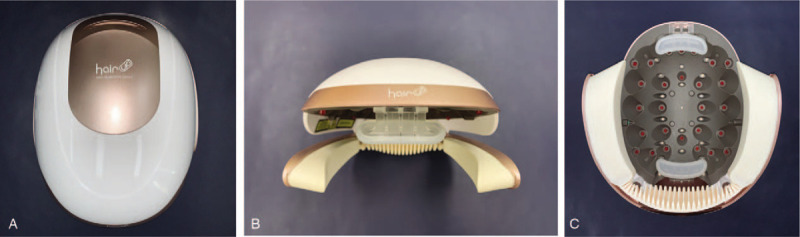
External appearance of the clinical devices. The experimental and sham devices had the same appearance. A. Upper surface, B. Front view, C. Inner surface.

Participants were randomly divided into 2 groups, which respectively used the experimental and sham helmet-type devices at home (Fig. 1). The experimental group was treated using a device containing a combination of a medical laser device and a low-level light irradiation device, composed of 21 LDs (wavelength, 655 ± 5 nm; mean output, 1.094 mW) and 30 LEDs (wavelength, 655 ± 20 nm; output, 1.75 mW). The sham device emitted only red therapeutic light from LED bulbs that were coated with red paint. The experimental devices emitted light with a mean output power of 2.56 mW/cm^2^ at a wavelength of 655 nm, and the control group was treated with a mean output power of 0 mW/cm^2^ (Table 2). Both devices were applied to the hair, including the area to be measured, for 25 minutes once every other day over the course of 16 weeks (in total: 56 times and 1,400 minutes). Before initiating the study, tattooing with blue dye was done at the central point of the vertex, as a reference point for accurate scalp measurements.

**Table 2:**
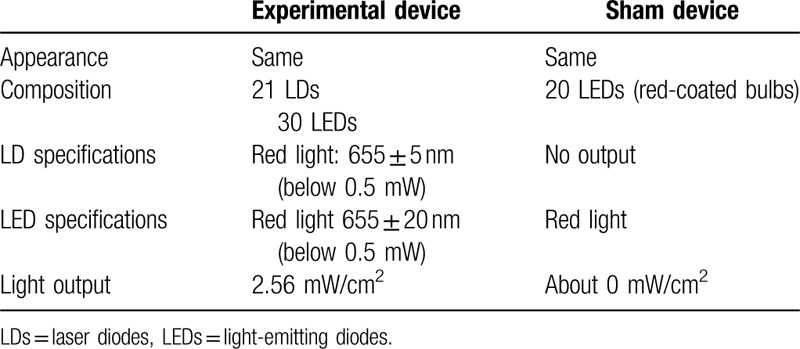
Comparison of the experimental device and sham device.

### 2.5 Efficacy evaluation

Subjects visited the medical center for evaluations at 0, 8, and 16 weeks (visit 1, visit 2, and visit 3). At the first visit, patients’ demographic information was obtained, and a physical examination was performed that included checking patients’ vital signs. Global photography and a phototrichogram were performed at baseline (screening test), as well as at 8 and 16 weeks. Participants’ satisfaction and perceived improvement were assessed at 8 and 16 weeks, and compliance with using the device was evaluated at visits 2 and 3.

#### 2.5.1 Primary endpoint

Phototrichogram assessment was done using a medical device (dpHarris Scalp & Hair Diagnosis System; Chowis company, Yongin-si, Gyeonggi-do, Republic of Korea) at baseline, 8 weeks, and 16 weeks. Measurements were performed through a × 50 magnification lens. Hair density (hairs/cm^2^) and the mean thickness (μm) of terminal hair were assessed by the clinical investigator at the tattooed region of each patient. We analyzed these 2 factors by evaluating the data obtained using a phototrichogram device. The primary endpoint of the study was defined as the difference in the rate of change of hair density between the test group and the control group. The secondary end point was the change of hair thickness.

#### 2.5.2 Patients’ satisfaction

A survey of subjective satisfaction was performed using a questionnaire containing 10 questions about improvements in hair loss at visit 2 and visit 3 (Table 3). Each item was measured on a 10-point scale (from 0 to 10), and total satisfaction was rated on a 100-point scale. The satisfaction scores of each group at visit 2 and visit 3 were compared in the statistical analysis.

**Table 3:**
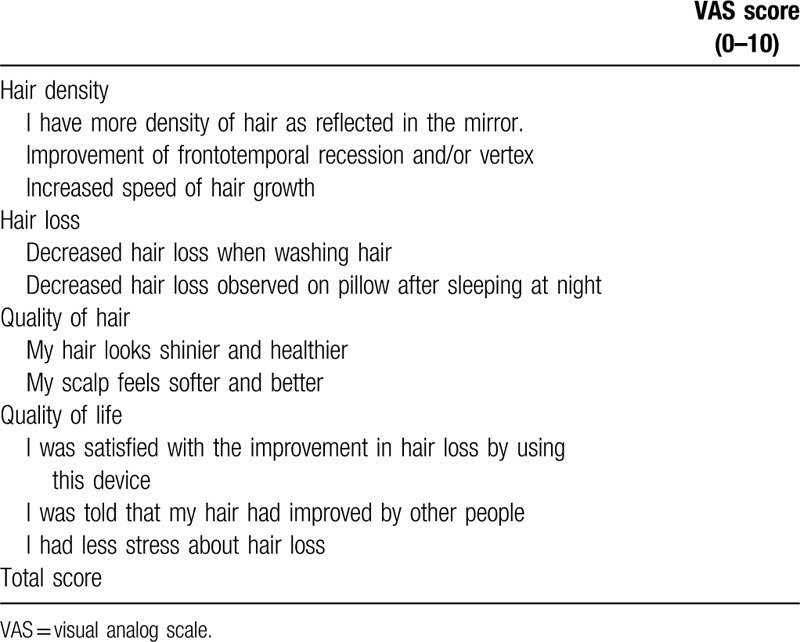
Questionnaire on subjective satisfaction.

#### 2.5.3 Patient compliance and adverse effects

Compliance was evaluated by checking the total hours of use and the number of experimental and sham devices provided to the subjects at visit 2 and visit 3. Participants who used the devices for less than 70% of what the protocol specified (assessed through both total hours and number of sessions) at each visit were excluded from this trial.

We investigated any discomfort and side effects at 8 weeks and 16 weeks. If an adverse symptom occurred, such as rash, swelling, itching, or irritation on the scalp, the medical device was immediately stopped. Candidates who reported an adverse effect were excluded and managed with appropriate treatment, regardless of clinical trial status.

### 2.6 Statistical analysis

The sample size was 60 patients, which was determined for analyzing the primary endpoint of improvement of hair density using PASS 14 (Power Analysis and Sample Size Software 2015; NCSS LLC, Kaysville, UT). This estimation was based on the data from the previous LLLT clinical trials performed by Lanzafame et al.^[21,22]^ All statistical analyses were conducted using SPSS version 18.0 (SPSS Inc., Chicago, IL). The demographic characteristics of the subjects at baseline were compared between the test and control groups. Quantitative data were presented as mean ± standard deviation and tested using the independent *t*-test, while categorical data were presented as frequency (%) and tested using the chi-square test or the Fisher exact test. To compare changes in hair density and hair thickness, the t-test and analysis of covariance were used, after adjusting for the following variables: age, sex, site, the intersection of age and site, and the intersection of sex and site. Satisfaction scores in both groups were compared by conducting analysis of covariance and repeated-measures analysis of variance after adjusting for the following variables: age, sex, and site. All statistical tests were performed using 2-sided tests, with the significance level set to *α*=0.05.

## 3 Results

Sixty patients, composed of 40 men and 20 women, participated in the trial at the 2 institutions. One male patient dropped out due to personal reasons and was excluded from the trial after the baseline visit, and a total of 59 patients were included in the final analysis of the results. The mean age was 49.55 years in the experimental group and 45.17 years in the sham device group (*P* = .181). There were no statistically significant differences at baseline between the groups in terms of demographic characteristics or hair loss-related characteristics such as hair loss classification, hair density, and hair thickness (Table 4).

**Table 4:**
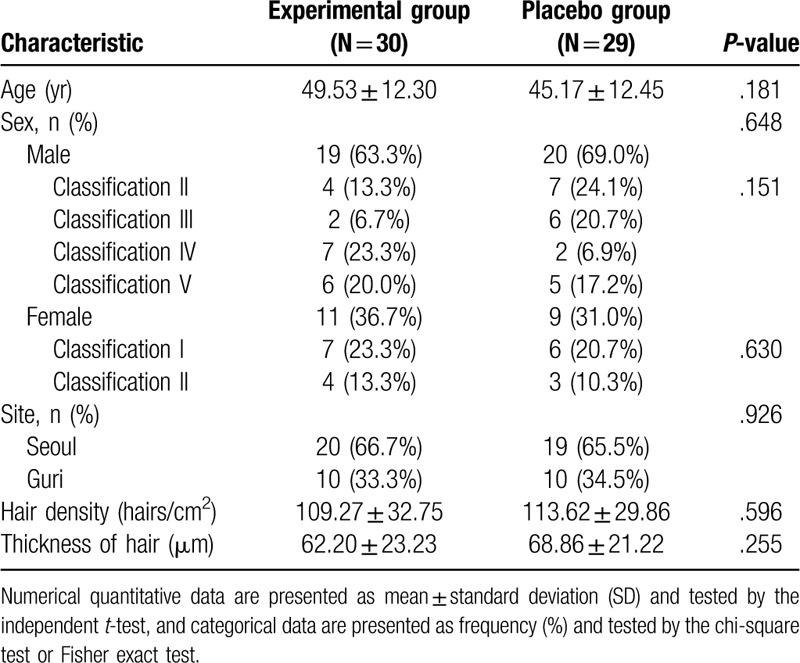
Baseline characteristics of study patients.

**Figure 2:**
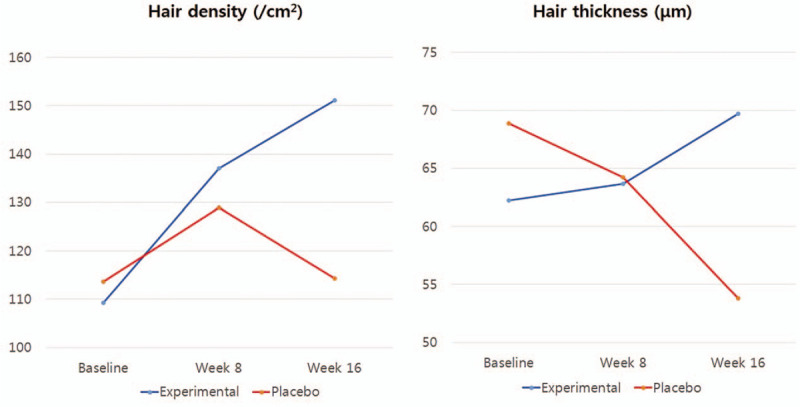
Hair density (hairs/cm^2^) and hair thickness (μm) at baseline, 8 weeks, and 16 weeks.

**Figure 3:**
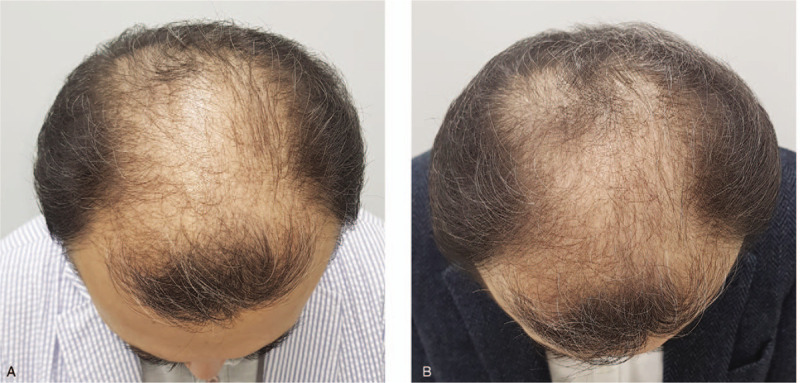
Hair vertex view of global photography of a 59-year-old male patient in the treatment group, diagnosed with Norwood-Hamilton type V hair loss. Compared to baseline, there was a 50% increase in hair density and a 14.9% increase in hair thickness at 16 wk. A. Baseline, B. 16 weeks.

**Figure 4:**
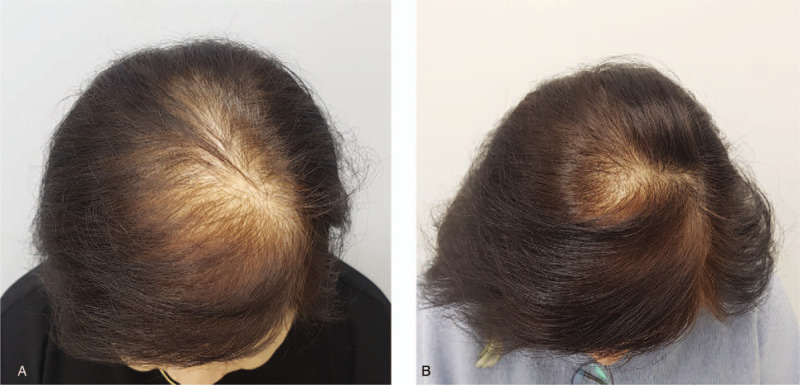
Hair vertex view of global photography of a 62-yr-old female patient in the treatment group, diagnosed with Ludwig type II hair loss. Compared to baseline, there was a 25% increase in hair density and a 3.1% increase in hair thickness at 16 wk. A. Baseline, B. 16 wk.

The average hair density and hair thickness of the terminal hair in the experimental group and sham device group were 109.27 hairs/cm^2^ versus 113.62 hairs/cm^2^ and 62.20 μm versus 68.86 μm at baseline, 137.03 hairs/cm^2^ versus 126.86 hairs/cm^2^ and 63.67 μm versus 64.21 μm at 8 weeks, and 151.17 hairs/cm^2^ versus 114.34 hairs/cm^2^ and 69.70 μm versus 53.83 μm at 16 weeks (Table 5, Fig. 2). Comparing the results at baseline and week 16, the hair density in the experimental group increased by 41.90 hairs/cm^2^ and the hair thickness increased by 7.50 μm, whereas the control group showed an increase of 0.72 hairs/cm^2^ and a decrease of 15.03 μm, respectively. For both endpoints, there were significant differences between the 2 groups (*P* < .001 for hair density, *P* < .001 for hair thickness) (Table 6). Global photography also confirmed a grossly visible increase in terminal hair density in the test group (Figs. 3 and 4).

**Table 5:**
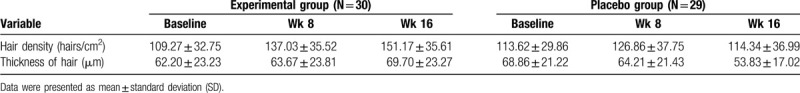
Measurements at baseline, 8 wk, and 16 wk according to the treatment.

**Table 6:**
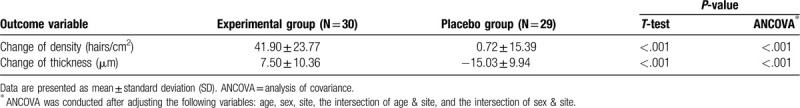
Comparison of outcomes after 16 wk between the treatment and placebo groups.

Participants completed satisfaction questionnaires at visits 2 and 3. In the experimental group and control group, the scores were 36.3 and 26.38 at 8 weeks and 43.17 and 35.14 points at 16 weeks, respectively. Satisfaction tended to be higher in the experimental group, but this trend did not reach statistical significance (*P* *=* .168 at 8 weeks, *P* *=* .265 at 16 weeks) (Table 7). All participants showed good compliance (over 70% of total use time and number of sessions at every visit). No adverse events or side effects occurred over the course of the 16-week clinical trial.

**Table 7:**
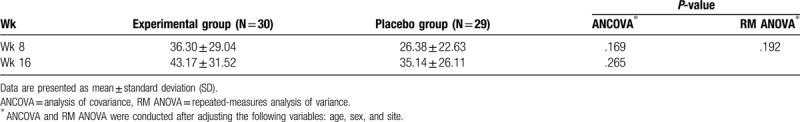
Comparison of satisfaction scores between the treatment and placebo groups.

## 4 Discussion

The prevalence of androgenetic alopecia has been reported to be lower in Asian populations than in Caucasian populations.^[17,18]^ In a 2001 study, the prevalence of androgenetic alopecia was 14.1% in men and 5.6% in women in South Korea.^[18]^ Treatment for androgenetic alopecia is also receiving increasing attention, especially as concerns about the aging process are growing. To date, 5-α reductase inhibitors, including finasteride and dutasteride, topical minoxidil, and combinations thereof are the most commonly used treatments for androgenetic alopecia. Some other complementary treatments exist, but those proven to be effective and safe are very limited. Furthermore. 5-α reductase inhibitors have the well-known side effects of sexual dysfunction and heart function problems in male patients, and minoxidil was also reported to have adverse effects such as contact dermatitis and facial hypertrichosis. There are few alternative treatment choices if patients experience side effects or do not respond to classical treatments.^[4–6]^

LLLT is a non-thermal light therapy method that has a photobiomodulatory effect. It has recently been used to promote wound healing, as well as for its anti-inflammatory properties, photo-rejuvenation, and photoprophylaxis, and as a treatment for various dermatologic disorders.^[7–9]^ In 1967, Endre Mester first observed that mice treated with lasers during experiments investigating the potential carcinogenic effects of laser exposure regrew hair in shaved areas significantly faster than unexposed mice.^[11]^ Subsequently. several studies—ranging from experimental animal studies to clinical studies—were conducted to show the effectiveness of LLLT for promoting hair growth, and LLLT was approved for hair loss treatment by the U.S. Food and Drug Administration in 2007.^[12–16]^ Although the exact mechanisms have not yet been identified, some cellular and molecular mechanisms have been identified in recent studies. LLLT is assumed to release nitric oxide from cytochrome c oxidase, a chromophore responsible for the absorption of red/infrared light, driving the electron transport chain to generate adenosine triphosphate and reactive oxygen species, as well as inducing transcription factors.^[19,20]^ As a result, it has been speculated that LLLT exerts its effects on hair growth by stimulating anagen re-entry in telogen hair follicles, prolonging the duration of the anagen phase, increasing the rate of proliferation in active anagen hair follicles, and preventing premature catagen development.^[19,20]^

The helmet-type light therapy device was simple and easy to use at home, and it had the advantage of being able to stimulate the entire scalp, making it easy to analyze its effects and making it suitable for use as a standard treatment. Significant improvements in hair density and hair thickness have been recorded in previous randomized controlled trials. Lanzafame et al conducted 2 randomized controlled trials in male and female patients using a helmet-type device delivering a 6-mW output at 655 nm for 16 weeks. Increased hair count (by 35% in male patients and 37% in female patients) was observed in the LLLT-treated group versus the sham-treated controls. ^[21,22]^ The study conducted by Kim et al used a 655-nm wavelength for 24 weeks, with 18 minutes of treatment daily. The hair density increased by a 17.2 hairs/cm^2^ and the hair thickness increased by 12.6 μm.^[23]^ In 2018, Mai-Yi Fan et al reported that the experimental group showed significant improvements, with hair count increasing by 6.7 hairs/cm^2^, hair thickness by 2.3 μm, and hair coverage by 2.4%.^[24]^ More recently, Sunchonwanit et al. conducted a similar study using a 5-mW output at a 660-nm wavelength for 24 weeks. They reported improvements in the hair count (10.21 hairs/cm^2^) and hair thickness (6.11 μm) in the laser group.^[25]^ There were no severe adverse effects in these studies, and only a few patients reported mild side effects such as headache, dry skin, pruritus, redness, or irritation at the targeted site.^[21–25]^

Only a few studies have been conducted of the efficiency and safety of helmet-type LLLT devices for the treatment of androgenetic alopecia, especially in Asian populations. In previous studies, the wavelength of 655 nm was proven to be most efficient for hair growth.^[12–15]^ Our helmet-type LLLT device contained a combination of 655-nm LDs and LEDs, and each used a continuous wave form with a maximum power of 5 mW, corresponding to about 2.36 mW/cm^2^. In order to maximize the accessibility of the treatment, we used a protocol in which the clinical device was used for 25 minutes every other day. In the experimental group, hair density and thickness increased by 41.90 hairs/cm^2^ and 7.50 μm, respectively. There was no significant change in hair density in the control group, although the hair thickness showed a marked reduction of 15.03 μm. This indicates that alopecia was progressing in the control group without treatment, and that LLLT prevented participants’ hair from changing to vellus hair. There were no statistically significant differences in subjective satisfaction, but slightly higher satisfaction was obtained in the experimental group. Based on the high compliance and low dropout rate of the clinical trial, we suggest that the helmet-type LLLT device was confirmed to be easy and convenient. No side effects and adverse reactions occurred in our study, as in previous studies.

Participants’ subjective satisfaction with their hair was assessed in this study. No significant difference in subjective satisfaction was found between the treatment group and the sham device group, although slightly higher scores were reported in the treatment group. This is similar to previous findings reported by Mai-Yi Fan et al and Kim et al ^[23,24]^ This inconsistency indicates that subjective satisfaction did not reflect the improvement in phototrichographic findings. These outcomes could have also stemmed from differences in personal expectations regarding the treatment of alopecia and limitations of our survey, such as its simplicity, the short-term follow-up period, and the relatively small sample size.

There are limitations to this trial, such as the short-term follow-up and the possibility of measurement bias, which could interfere with accurate counting and analysis. In this study, we only collected calculated data from the phototrichogram device after analyzing the photos which were captured in real time, which may have contributed to measurement bias as a limitation. Furthermore, the possibility cannot be excluded that patients may have received other hair care and hair loss treatments during the clinical trial. We suggest that further studies should include a long-term follow-up to evaluate long-term outcomes and side effects. Additionally, differences in efficiency according to age and the stage of hair loss need to be compared and analyzed. Furthermore, research into the efficacy of combining LLLT with classic treatments such as 5-α reductase inhibitors and/or minoxidil should be conducted.

## 5 Conclusion

In this study, a helmet-type LLLT device showed a significant effect on increasing hair density and hair thickness in androgenetic alopecia in both sexes. Therefore, LLLT could be a safe and effective alternative monotherapy for androgenetic alopecia.

## Acknowledgments:

The authors would like to thank Prof. Nam Eun Woo (Medical Statistics Consulting Service) for the statistical consultation.

## Author contributions

**Conceptualization:** Jung Soo Yoon, Jang Hyun Lee, Hee Chang Ahn.

**Data curation:** Jung Soo Yoon, Won Young Ku.

**Formal analysis:** Jung Soo Yoon.

**Funding acquisition:** Hee Chang Ahn.

**Investigation:** Jung Soo Yoon, Won Young Ku.

**Methodology:** Hee Chang Ahn.

**Project administration:** Jang Hyun Lee, Hee Chang Ahn.

**Supervision:** Jang Hyun Lee, Hee Chang Ahn.

**Writing – original draft:** Jung Soo Yoon, Won Young Ku.

**Writing – review & editing:** Jang Hyun Lee, Hee Chang Ahn.

## References

[1] Bergfeld WF Androgenetic alopecia: an autosomal dominant disorder. Am J Med 1995;98 (1A):95S–8S. 7825647 10.1016/s0002-9343(99)80065-5

[2] Olsen EA Messenger AG Shapiro J Evaluation and treatment of male and female pattern hair loss. J Am Acad Dermatol 2005;52:301–11. 15692478 10.1016/j.jaad.2004.04.008

[3] Gupta M Mysore V Classifications of patterned hair loss: a review. J Cutan Aesthet Surg 2016;9:3–12. 27081243 10.4103/0974-2077.178536 PMC4812885

[4] Bienová M Kucerová R Fiurásková M Androgenetic alopecia and current methods of treatment. Acta Dermatovenerol Alp Pannonica Adriat 2005;14:5–8. 15818439

[5] Varothai S Bergfeld WF Androgenetic alopecia: an evidence-based treatment update. Am J Clin Dermatol 2014;15:217–30. 24848508 10.1007/s40257-014-0077-5

[6] Rossi A Anzalone A Fortuna MC Multi-therapies in androgenetic alopecia: review and clinical experiences. Dermatol Ther 2016;29:424–32. 27424565 10.1111/dth.12390

[7] Jagdeo J Austin E Mamalis A Light-emitting diodes in dermatology: a systematic review of randomized controlled trials. Lasers Surg Med 2018;50:613–28. 10.1002/lsm.22791 PMC6099480 29356026

[8] Barolet D Light-emitting diodes (LEDs) in dermatology. Semin Cutan Med Surg 2008;27:227–38. 19150294 10.1016/j.sder.2008.08.003

[9] Avci P Gupta A Sadasivam M Low-level laser (light) therapy (LLLT) in skin: stimulating, healing, restoring. Semin Cutan Med Surg 2013;32:41–52. 24049929 PMC4126803

[10] Juhász ML Levin MK Marmur ES A review of available laser and intense light source home devices: a dermatologist's perspective. J Cosmet Dermatol 2017;16:438–43. 28741866 10.1111/jocd.12371

[11] Mester E Szende B Gartner P The effect of laser beams on the growth of hair in mice (German). Radiobiol Radiother (Berl) 1968;9:621–6. 5732466

[12] Wikramanayake TC Villasante AC Mauro LM Low-level laser treatment accelerated hair regrowth in a rat model of chemotherapy-induced alopecia (CIA). Lasers Med Sci 2013;28:701–6. 22696077 10.1007/s10103-012-1139-7

[13] Afifi L Maranda EL Zarei M Low-level laser therapy as a treatment for androgenetic alopecia. Lasers Surg Med 2017;49:27–39. 27114071 10.1002/lsm.22512

[14] Liu KH Liu D Chen YT Comparative effectiveness of low-level laser therapy for adult androgenic alopecia: a system review and meta-analysis of randomized controlled trials. Lasers Med Sci 2019;34:1063–9. 30706177 10.1007/s10103-019-02723-6

[15] Darwin E Heyes A Hirt PA Low-level laser therapy for the treatment of androgenic alopecia: a review. Lasers Med Sci 2018;33:425–34. 29270707 10.1007/s10103-017-2385-5

[16] Najem I Chen H Use of low-level laser therapy in treatment of the androgenic alopecia, the first systematic review. J Cosmet Laser Ther 2018;20:252–7. 29227728 10.1080/14764172.2017.1400174

[17] Wang TL Zhou C Shen YW Prevalence of androgenetic alopecia in China: a community-based study in six cities. Br J Dermatol 2010;162:843–7. 20105167 10.1111/j.1365-2133.2010.09640.x

[18] Paik JH Yoon JB Sim WY The prevalence and types of androgenetic alopecia in Korean men and women. Br J Dermatol 2001;145:95–9. 11453914 10.1046/j.1365-2133.2001.04289.x

[19] Chung H Dai T Sharma SK The nuts and bolts of low-level laser (light) therapy. Ann Biomed Eng 2012;40:516–33. 22045511 10.1007/s10439-011-0454-7 PMC3288797

[20] de Freitas LF Hamblin MR Proposed MECHANISMS OF PHOTOBIOMODULATION OR LOW-LEVEL LIGHT THERAPY. IEEE J Sel Top Quantum Electron 2016;22 (3.):10.1109/JSTQE.2016.2561201 PMC5215870 28070154

[21] Lanzafame RJ Blanche RR Bodian AB The growth of human scalp hair mediated by visible red light laser and LED sources in males. Lasers Surg Med 2013;45:487–95. 24078483 10.1002/lsm.22173

[22] Lanzafame RJ Blanche RR Chiacchierini RP The growth of human scalp hair in females using visible red light laser and LED sources. Lasers Surg Med 2014;46:601–7. 25124964 10.1002/lsm.22277 PMC4265291

[23] Kim H Choi JW Kim JY Low level light therapy for androgenetic alopecia: a 24-week, randomized, double-blind. Sham device-controlled multicenter trial. Dermatol Surg 2013;39:1177–83. 23551662 10.1111/dsu.12200

[24] Mai-Yi Fan S Cheng YP Lee MY Efficacy and safety of a low-level light therapy for androgenetic alopecia: a 24-week, randomized, double-blind, self-comparison. Dermatol Surg 2018;44:1411–20. 29957664 10.1097/DSS.0000000000001577

[25] Suchonwanit P Chalermroj N Khunkhet S Low-level laser therapy for the treatment of androgenetic alopecia in Thai men and women: a 24-week, randomized, double-blind, sham device-controlled trial. Lasers Med Sci 2019;34:1107–14. 30569416 10.1007/s10103-018-02699-9

